# Gender equity and professional experience of female colorectal surgeons in Italy: results of a National survey

**DOI:** 10.1007/s13304-026-02622-w

**Published:** 2026-04-04

**Authors:** Roberta Tutino, Luca Manfrino, Simona Deidda, Gaya Spolverato, Roberto Passera, Isabella Frigerio, Laura Lorenzon, Daniela Rega, Paola De Nardi

**Affiliations:** 1 Department of Surgical Sciences, University of Turin, Turin, Italy; 2 Colorectal Surgery, IRCSS San Raffaele Scientific Institute, Milan, Italy; 3 Department of Surgical Science, Colorectal Surgery Center, University of Cagliari, Cagliari, Italy; 4 Department of Surgery, Oncology and Gastroenterology University Hospital of Padova, Padua, Italy; 5 Department of Medical Sciences, University of Turin, Turin, Italy; 6 Hepatobiliary and Pancreatic Surgery, Pederzoli Hospital- Peschiera del Garda, Peschiera del Garda, Italy; 7 Collegium Medicum, SAN University, Lodz, Poland; 8 Fondazione Policlinico Universitario A. Gemelli IRCCS - Rome, Rome, Italy; 9 Division of Colorectal Surgical Oncology, Department of Abdominal Oncology, Istituto Nazionale Tumori IRCCS “Fondazione G. Pascale”, Naples, Italy

**Keywords:** Colorectal surgery, Gender equity, Job satisfaction, Mentoring, Work-life balance

## Abstract

Gender disparities in surgical careers are increasingly recognized in both academic research and everyday clinical practice. Although women now represent a substantial proportion of medical graduates and surgical trainees, they remain underrepresented in surgical specialties, particularly in technically demanding and high-stress fields such as colorectal surgery. In Italy, Women in Surgery (WIS) has previously explored gender dynamics among surgeons in general. The aim of the present study was to investigate the professional experience of female colorectal surgeons. A national, anonymous, web-based survey was conducted as a joint initiative of Women in Surgery Italia (WIS) and the Italian Society of Colorectal Surgery (SICCR). The questionnaire was developed, reviewed, and finalized collaboratively by members of both societies. A total of 119 female surgeons completed the survey. Operative exposure was limited, and perceptions of gender bias and microaggressions were common. Ergonomic discomfort and challenges related to maternity reintegration were frequently reported. This study provides the first national overview of the professional experiences of female colorectal surgeons in Italy and highlights persistent structural and cultural barriers affecting career development.

**Supplementary Information:**

The online version contains supplementary material available at 10.1007/s13304-026-02622-w.

## Introduction

Gender disparities in surgical careers are increasingly recognized in both academic research and everyday clinical practice [1, 2]. Although women now represent a substantial proportion of medical graduates and surgical trainees, they remain underrepresented in surgical specialties, particularly in technically demanding and high-stress fields such as colorectal surgery [3–5].

Previous international studies have highlighted persistent gaps in operative exposure, access to leadership roles, mentorship opportunities, and perceptions of equity within surgical environments [1, 6]. These disparities have tangible consequences not only for individual career development, but also for workforce sustainability, diversity, and innovation within surgery.

In Italy, Women in Surgery (WIS) has previously explored gender dynamics among surgeons in general. However, the professional experience of female colorectal and proctologic surgeons has never been specifically investigated. This subgroup faces unique challenges related to workload intensity, oncologic responsibility, procedural complexity, and prolonged training pathways [7, 8].

To address this gap, we conducted a national survey focusing on operative exposure, workplace dynamics, ergonomics, and the impact of maternity on professional trajectories. To our knowledge, this represents the first national study specifically examining the experiences of female colorectal and proctologic surgeons in Italy.

## Methods

A national, anonymous, web-based survey was conducted as a joint initiative of Women in Surgery Italia (WIS) and the Italian Society of Colorectal Surgery (SICCR). The questionnaire was developed, reviewed, and finalized collaboratively by members of both societies.

Eligible participants were female surgeons or surgical trainees practicing colorectal or proctologic surgery in Italy. Non-surgical specialists and male surgeons were excluded.

The survey consisted of 41 items addressing demographics and training background, workload and surgical exposure, case assignment and career opportunities, gender bias and microaggressions, ergonomics and instrument suitability, and maternity leave and reintegration. Responses included Likert-scale items, multiple-choice questions, and optional open-ended comments. Questionnaire is fully provided in the supplement.

The survey was distributed through the WIS mailing list, the SICCR mailing list, and professional colorectal surgery networks. Data collection took place between December 2024 and February 2025. Participation was voluntary and anonymous.

### Statistical analysis

Quantitative data were described as medians and interquartile range (IQR), while categorical variables were listed as absolute and relative frequencies. The association between the variables of interest was estimated by the Fisher’s exact test. The statistical significance level was 0.05 (two-sided). The analyses were executed in R (version 4.5.1). No imputation of missing data was performed.

## Results

### Participants and profile

A total of 119 female surgeons completed the survey. Thirty-nine respondents were younger than 35 years, 45 were between 35 and 45 years, and 35 were older than 45 years. Twenty-four participants were trainees. Most respondents were general surgeons with a colorectal emphasis (74) followed by colorectal specialists (17), colorectal/proctologic surgeons (20) and proctologists (8). The majority were employed in public hospitals (104). Regarding employment status, 86 participants held fixed-term contracts, 23 reported precarious contracts, and 10 were self-employed. In most surgical units, fewer than five women were present (83 units), and 14 respondents reported being the only woman in their team. In 78 units, the female-to-male ratio was approximately 30%. (Table 1).

**Table 1:** Demographic and professional characteristics of the participants

Item		n	%
Age	< 35	38	32
	35–45	47	39.5
	> 45	34	28.6
Professional role	General surgeons	71	62
	Dedicated colorectal surgeons	16	14
	Colorectal/proctologists	20	17
	Proctologists only	8	7
Workplace	Public	99	85.7
	Private	16	14.3
Employment status	Permanent	85	71.4
	Fixed-term	26	21.8
	Self-employed	8	6.7
N. of female in the surgical team	1	14	12
	< 5	80	70
	> 5	20	18
Female/male ratio	< 30%	24	21
	30–70%	76	66
	> 70%	15	13

### Workload and exposure to operations

Night and holiday duties were commonly reported. Thirty-three respondents spent more than 60% of their working time in the operating room, whereas 36 spent less than 30%. In 60 respondents, non-operative activities accounted for 30–60% of working time. Institutional surgical volumes varied widely. Twenty-four centers reported performing more than five major colorectal procedures per week, whereas 91 reported fewer than five, including 37 centers performing fewer than two. At an individual level, 52 respondents acted as first or second surgeon in fewer than 30% of cases, and 82 were first surgeon in fewer than 10% of major colorectal procedures (Table 2).

**Table 2:** Workload and surgical activity in the OR

Item		n	%
Working hours	< 39	24	20.2
	40–49	41	34.5
	> 50	54	45.4
% of oupatients clinics and ward working hours	< 30%	27	22.7
	30–60%	56	48.7
	> 60%	32	28.6
Surgical involvement (% of participation as first/second surgeons)	< 30%	51	44
	30–60%	35	31
	> 60%	29	25
% tutoring	< 30%	77	86
	30–60%	0	0
	> 60	13	14
n. colorectal surgeries per week in the unit	< 2	37	32.2
	2–5	54	46.9
	> 5	24	20.9

### Career satisfaction and assignment of cases

Sixty respondents reported partial satisfaction with their surgical activity, while 27 were dissatisfied. The remaining respondents reported full satisfaction or did not provide a definite answer. Limited operative opportunities and lack of protected surgical time were the most frequently cited reasons for dissatisfaction. Case assignment was perceived to be influenced by technical competence, personal relationships with senior staff, and availability. Several respondents also reported experiences of sexism, hazing, or restricted access to the operating room. Teaching involvement was limited, with 102 participants spending less than 30% of their working time on tutoring or mentoring activities.

### Perceived gender bias and microaggressions

**Fig. 1:**
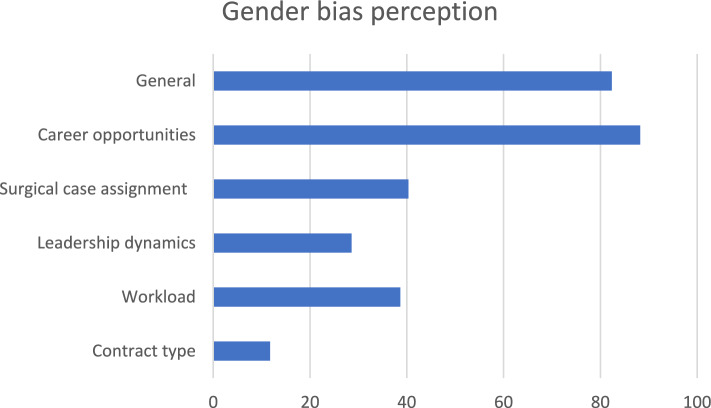
Gender bias

**Fig. 2:**
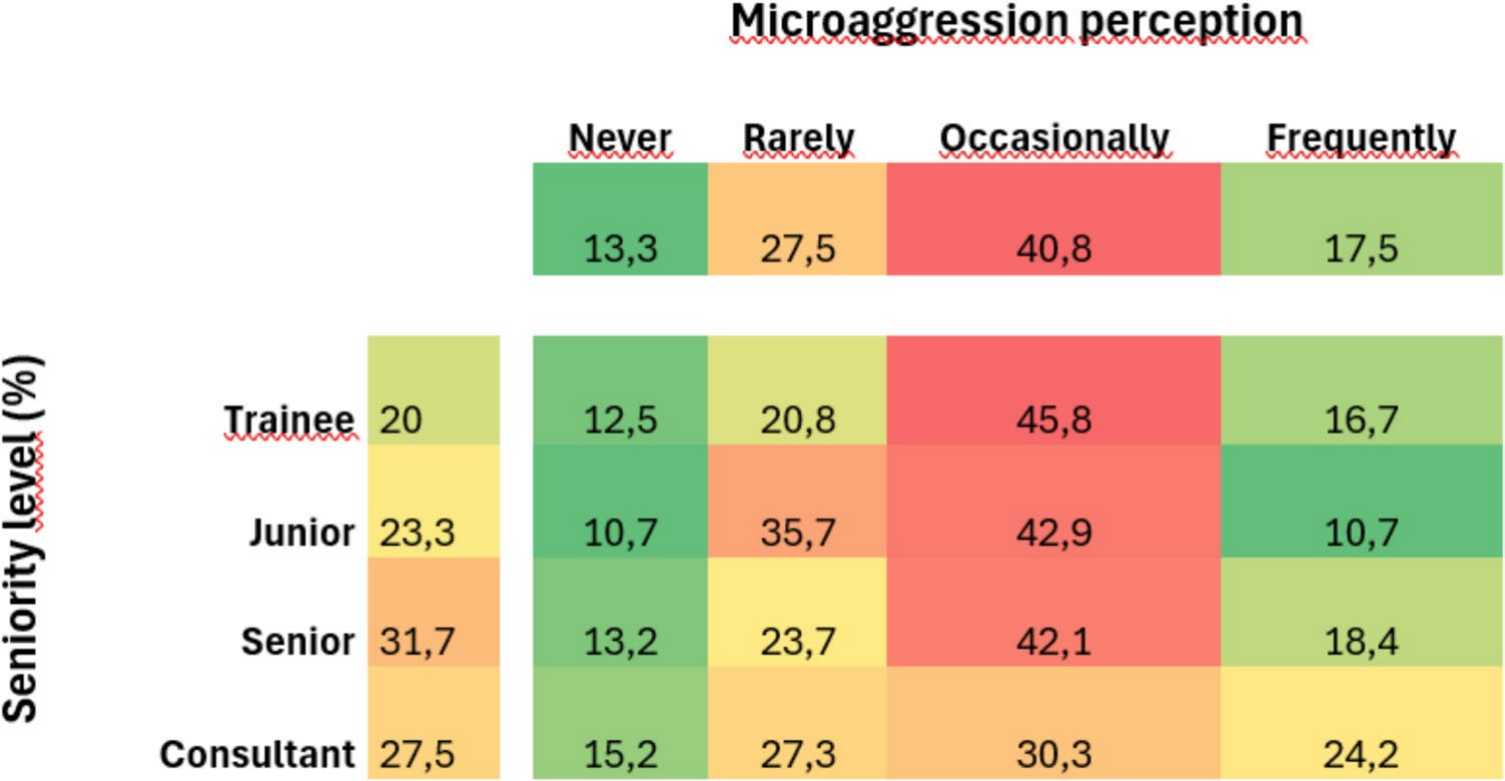
Heatmap: Microaggressions vs Seniority

Gender-based contractual differences were reported by 14 respondents. A total of 109 participants believed that gender influenced career advancement. (Fig. 1) Differential treatment compared with male colleagues was reported by 92 respondents, particularly regarding access to complex cases, training opportunities, career advancement, and workload distribution. Microaggressions were common. Twenty respondents reported never experiencing them, whereas 19 reported frequent episodes. Most respondents described occasional experiences. (Fig. 2) Common examples included: doubts about technical ability, comments related to gender roles, paternalistic behaviour, exclusion from opportunities, occasional sexual innuendo. Overall, 92 participants reported that these behaviours negatively affected the work environment.

### Workplace infrastructure and ergonomics

Inadequate rest facilities were reported by 67 respondents. Ergonomic discomfort with standard surgical instruments was widespread: only 21 respondents felt instruments were adequately sized, while 109 reported difficulties related to ergonomics.

### Parenthood and reintegration

Seventy-five respondents had never been pregnant. Among the 44 who had experienced pregnancy, 26 reported delayed return to full duties after maternity leave, and 5 reported no structured or progressive reintegration.

### Suggested interventions

Respondents most frequently indicated the need for improved work–life balance (86), leadership opportunities (76), maternity and career policies (68), transparent promotion criteria (67), anti-sexism initiatives (52), and increased visibility at scientific meetings (48). (Table 3).

**Table 3:** Needs and suggested interventions

Intervention type	% endorsement
*Needs*	
Work/life balance support	72
More leadership opportunities	64
Policies supporting maternity and equal career advancement	57
Fair career advancement	56
Anti-sexism initiatives	44
Greater visibility at conferences	40
*Suggested interventions*	
Maternity support and reintegration policies	59
Promote female leadership	55
Implement fair assessment of skills	56
Support work-life balance	44
Offer continuous training with a focus on inclusivity	29
Develop specific mentorship programs for women	26

**Fig. 3:**
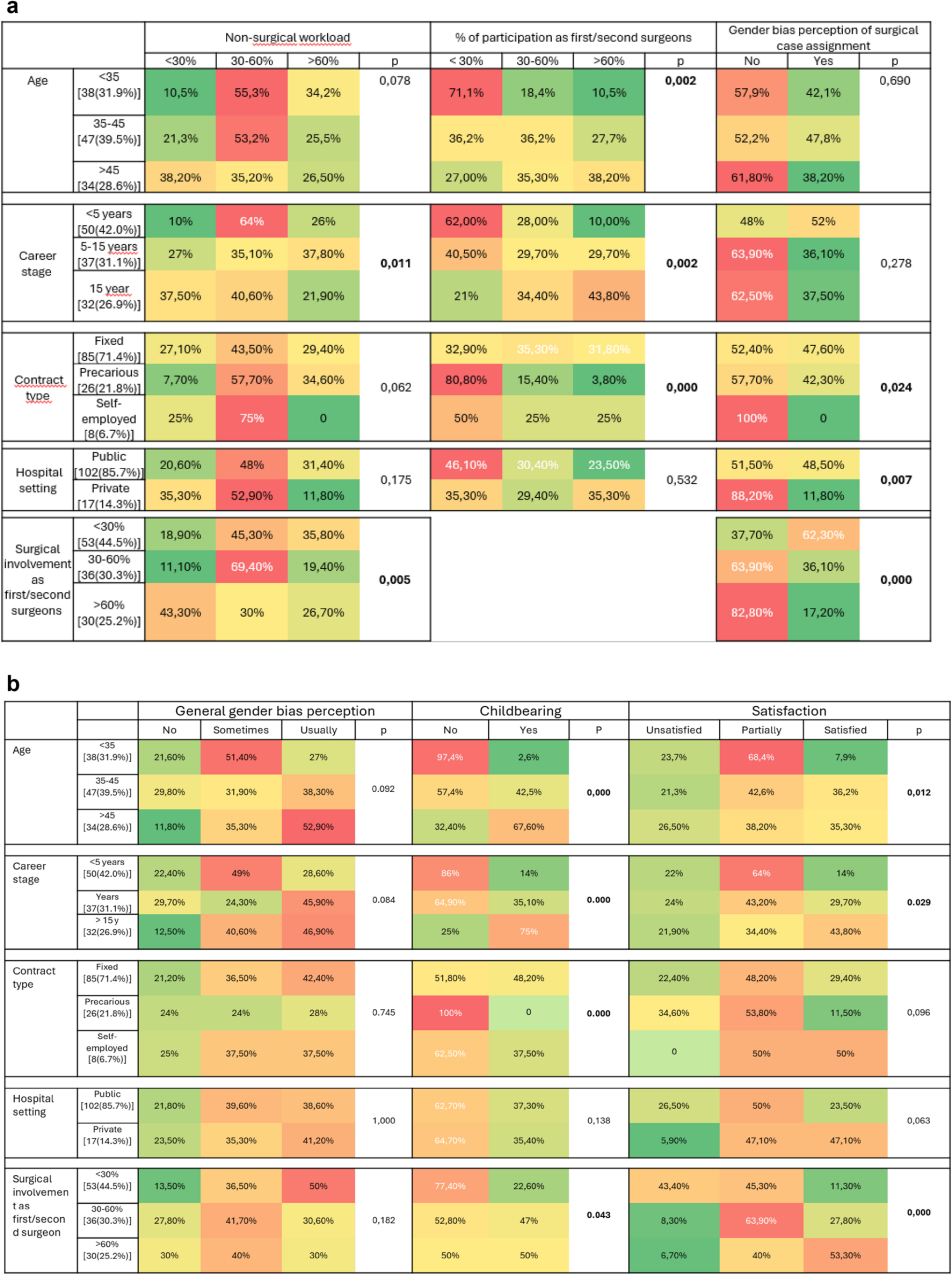
**a** Stratified analysis by age, career stage, contract type, hospital setting, and surgical involvement. **b** Stratified analysis by age, career stage, contract type, hospital setting and surgical involvement

Stratified analyses by age, career stage, contract type, hospital setting, and surgical involvement revealed significant differences in workload distribution, operative roles, perception of gender bias, and childbearing. (Fig. 3a and b).

Participation as first or second surgeon increased with age (*p* = 0.002). Satisfaction increased with greater surgical involvement (*p* < 0.001). Higher operative exposure was associated with lower perceived gender bias in the assignment of surgical cases (*p* < 0.001) and a reduced impact of harassment on work experience (*p* = 0.006). Childbearing increased with age and was inversely associated with operative involvement (*p* = 0.043).

## Discussion

This study provides the first national overview of the professional experiences of female colorectal surgeons in Italy and highlights persistent structural and cultural barriers affecting career development.

Job stability did not translate into operative opportunity. Although most respondents held stable contracts, only a minority regularly performed major colorectal procedures as first surgeons.

Case allocation appeared to be influenced by informal hierarchies and interpersonal dynamics rather than transparent, merit-based criteria.

Greater operative involvement was consistently associated with higher professional satisfaction, reduced perception of gender bias, lower reported impact of harassment, and a higher likelihood of childbearing.

Nevertheless, only a limited proportion of respondents reported substantial operative exposure, underscoring a gap between potential and opportunity.

Microaggressions were frequently encountered and perceived as detrimental to the work environment. Behaviors such as paternalism, doubts about technical competence, and exclusion from opportunities were commonly reported and align with recent national evidence on gender-based discrimination in surgical settings [9].

Work–life balance and parenthood emerged as central issues. The high number of childless surgeons and the difficulties reported after maternity leave suggest the presence of structural barriers that may contribute to attrition, as described in other surgical specialties [10–14].

Ergonomics represents an underrecognized but relevant issue. Standard surgical instruments often fail to accommodate anthropometric differences, potentially affecting performance, comfort, and long-term musculoskeletal health. Addressing this aspect represents a tangible opportunity for innovation.

Finally, limited involvement of women in tutoring and mentoring roles may contribute to the scarcity of visible role models, influencing career choices among trainees [15–17]. Structured mentorship programs tailored to specific professional needs may represent an effective strategy to promote gender equity and career advancement in colorectal surgery [18, 19].

In this context, initiatives such as the Mentor-WIS program, which pairs mentors and mentees according to specific professional needs may represent an effective model to support career advancement and promote gender equity in surgery.

This study has limitations. The number of respondents is representative of the small national population of female colorectal surgeons. Participation was voluntary and may be subject to self-selection. There are other implications too: the survey focused on clinical activity in the hospital and did not include academic productivity, which requires further analysis.

## Conclusion

In conclusion, this is the first national overview of the experiences of female colorectal surgeons in Italy. The survey underlines critical issues about operative exposure, maternity and reintegration, work-life balance, workplace dynamics, ergonomics, and sexism that affect career development and retention in high-complexity surgical fields. To fill these gaps, coordinated institutional and policy actions are needed, including transparent skill assessment, equal opportunities for career advancement, inclusive continuous training, and the establishment of specific mentorship programs for women. These priorities are consistent with international evidence and emphasize that systemic changes are urgently needed to ensure that female surgeons have the chance to fully develop their professional potential.

## Supplementary Information

Below is the link to the electronic supplementary material.

Supplementary file1 (XLSX 285 KB)

## Data Availability

The data that support the fi ndings of this study are available from the corresponding author upon request.

## Declarations

### CROSS statement

The authors have read the CROSS statement, and the manuscript was prepared and revised according to the CROSS statement.
